# *PCSK1* rs6232 Is Associated with Childhood and Adult Class III Obesity in the Mexican Population

**DOI:** 10.1371/journal.pone.0039037

**Published:** 2012-06-21

**Authors:** Marisela Villalobos-Comparán, Hugo Villamil-Ramírez, Teresa Villarreal-Molina, Elena Larrieta-Carrasco, Paola León-Mimila, Sandra Romero-Hidalgo, Leonor Jacobo-Albavera, Adriana E. Liceaga-Fuentes, Francisco J. Campos-Pérez, Blanca E. López-Contreras, Teresa Tusié-Luna, Blanca E. del Río-Navarro, Carlos A. Aguilar-Salinas, Samuel Canizales-Quinteros

**Affiliations:** 1 Departamento de Biología, Facultad de Química, Universidad Nacional Autónoma de México (UNAM), Mexico City, Mexico; 2 Unidad de Biología Molecular y Medicina Genómica, Instituto Nacional de Ciencias Médicas y Nutrición “Salvador Zubirán” (INCMNSZ), Mexico City, Mexico; 3 Instituto Nacional de Medicina Genómica (INMEGEN), Mexico City, Mexico; 4 Clínica de Obesidad, Hospital Rubén Leñero, Mexico City, Mexico; 5 Instituto de investigaciones Biomédicas, Universidad Nacional Autónoma de México (UNAM), Mexico City, Mexico; 6 Departamento de Alergia e Inmunología Clínica, Hospital Infantil de México Federico Gómez, Mexico City, Mexico; 7 Departmento de Endocrinología y Metabolismo, INCMNSZ, Mexico City, Mexico; Tulane School of Public Health and Tropical Medicine, United States of America

## Abstract

**Background:**

Common variants rs6232 and rs6235 in the *PCSK1* gene have been associated with obesity in European populations. We aimed to evaluate the contribution of these variants to obesity and related traits in Mexican children and adults.

**Methodology/Principal Findings:**

Rs6232 and rs6235 were genotyped in 2382 individuals, 1206 children and 1176 adults. Minor allele frequencies were 0.78% for rs6232 and 19.99% for rs6235. Rs6232 was significantly associated with childhood obesity and adult class III obesity (OR = 3.01 95%CI 1.64–5.53; *P* = 4×10^−4^ in the combined analysis). In addition, this SNP was significantly associated with lower fasting glucose levels (*P* = 0.01) and with increased insulin levels and HOMA-B (*P* = 0.05 and 0.01, respectively) only in non-obese children. In contrast, rs6235 showed no significant association with obesity or with glucose homeostasis parameters in any group.

**Conclusion/Significance:**

Although rs6232 is rare in the Mexican population, it should be considered as an important risk factor for extreme forms of obesity.

## Introduction

The prevalence of obesity has increased worldwide, including Mexico, where more than 70% of the adults and 26% of the children are overweight or obese [1]. Even though obesity has a strong genetic contribution, the identification of genes related to obesity risk has proven difficult [2]–[4]. The discovery of genes causing monogenic forms of obesity such as the prohormone convertase subtilisin/kexin type 1 gene (*PCSK1*) has greatly improved our understanding of the pathophysiology of obesity [2]–[4]. This gene encodes an enzyme expressed in neuroendocrine cells that converts inactive prohormones into functional key hormones that regulate central and/or peripheral energy metabolism. Although loss-of-function mutations in this gene causing childhood obesity and impaired glucose tolerance are rare [5]–[7], two common nonsynonymous variants (rs6232 and rs6235) were recently found to be strongly associated with childhood and adulthood obesity in European population [8]. Functional *in vitro* analysis of these variants revealed a significant impairment of the catalytic activity of the enzyme for rs6232 (N221D), but no enzyme activity alteration for rs6235 (S690T) [8].

Several studies have sought to replicate the association of these variants (mainly rs6235) with obesity and obesity-associated traits in Asian and European adult populations, with inconsistent results [9]–[15]. These population-based studies included only a reduced number of obese class III individuals and did not include children as the initial report of Benzinou *et al*. [8]. This may partially explain such inconsistencies, as genetic influences on BMI may be stronger precisely in children and individuals with class III obesity [2], [16]. Thus, the aim of the present study was to analyze the association of rs6232 and rs6235 with obesity and related traits in a case-control analysis of Mexican-Mestizo adults and children.

## Materials and Methods

### Subjects

The study included 1206 non-related Mexican-Mestizo children aged 5 to 12 years (596 boys and 610 girls), recruited at a summer camp for children of employees of the Mexican Health Ministry (Convivencia Infantil 2008–2009, Secretaría de Salud) and the Hospital Infantil de México. Body mass index (BMI) was calculated as weight in kilograms divided by height in meter squared. BMI z-scores and percentiles were calculated using age and sex specific BMI reference data, as recommended by the Centers for Disease Control and Prevention [17]. The population was stratified based on percentile BMI, 802 children were non-obese (BMI percentile<95^th^) and 404 were obese (BMI percentile≥95^th^).

The study also included 1176 unrelated Mexican Mestizos aged 18–82 years (806 nonpregnant women and 370 men): 788 individuals described by Villalobos-Comparán et al. [18] and 388 additional subjects recruited from Obesity Clinics at the INCMNSZ and Ruben Leñero Hospital. Two hundred and fifty four (21.59%) of these individuals had been diagnosed with type 2 diabetes (T2D) according to WHO (World Health Organization) criteria [19]. Individuals were grouped according to BMI: 562 were non-obese (BMI <30 kg/m^2^), 380 had class I/II obesity (30≤ BMI <40 kg/m^2^), and 234 had class III obesity (BMI ≥40 kg/m^2^). Biochemical parameters were measured in blood samples obtained after 12-h fast as previously described [20]. Homeostasis model assessment of beta-cell function (HOMA-B) and insulin sensitivity (HOMA-S) as measures of beta-cell function and insulin sensitivity were estimated using a computer model [21]. The characteristics of the children and adult populations are shown in Table 1.

**Table 1:** Characteristics of the cases and controls in the children and adult populations.

Children			
		Non-obese	Obese
*N (1206)*		802	404
Males (%)		354 (44.13)	236 (58.41)
Age (yrs)		9.40±1.92	10.25±2.27
z-score BMI		0.31±1.00	2.08±0.28
Glucose (mmol/L)		5.00±0.45	5.01±0.52
Insulin (pmol/L)		39.5 (23.6, 59.0)	76.3 (48.6, 108.3)
HOMA-B (%)		76.3 (57.1, 102.7)	125.2 (84.8, 158.7)
HOMA-S (%)		136.5 (90.7, 222.1)	69.1 (49.8, 107.6)
**Adults**			
*N (1176)*	562		614
Males (%)	191 (33.98)		179 (29.15)
Age (yrs)	47.40±14.73		41.91±12.68
BMI (Kg/m^2^)	24.20±2.67		38.51±7.65
Glucose (mmol/L)	5.88±2.44		6.20±2.46
Insulin (pmol/L)	61.1 (39.5, 104.1)		75.0 (45.1, 123.6)
HOMA-B (%)	93.7 (66.3, 131.4)		99.8 (61.2, 136.0)
HOMA-S (%)	83.2 (50.3,130.5)		71.0 (41.2, 116.8)

Data are means ± s.d. or medians (interquantile range). HOMA-B, homeostasis model assessment of beta-cell function; HOMA-S, homeostasis model assessment of insulin sensitivity.

As part of the eligibility criteria, subjects with thyroid gland disease or showing weight instability three months prior to the study were excluded, as well as elderly subjects with signs of dementia by Mini-Mental state examination [22]. Only individuals born in Mexico whose parents and grandparents identified themselves as Mexican Mestizos were included. This project was approved by the Institutional Committee of Biomedical Research in Humans of the INCMNSZ. All adult participants and parents of the children provided written informed consent prior to their inclusion in the study.

### Single Nucleotide Polymorphism Genotyping

Rs6232 and rs6235 were genotyped using TaqMan assays (ABI Prism 7900HT Sequence Detection System; Applied Biosystems, Foster City, CA). Genotyping call rate exceeded 97% per SNP and no discordant genotypes were observed in 25 duplicate samples. In addition, because the Mexican-Mestizo population is admixed, we analyzed 10 ancestry informative markers to rule out population stratification [18]. Genotyping was performed by KBiosciences (Hertfordshire, UK, http://www.kbioscience.co.uk/) using a KASPar assay system. Genotyping call rates of each ancestry informative marker exceeded 95%, and no discordant genotypes were observed in 54 duplicate samples. Deviation from Hardy–Weinberg equilibrium was not observed for rs6232 and rs6235 in any group (*P*>0.66 and *P*>0.21, respectively).

### Statistical Analysis

Logistic regression was used to test for associations between the rs6232 and rs6235 SNPs and obesity. Children and adult combined odds ratios were estimated using the Mantel-Haenszel method. The AdmixMap program was used to test the possible effect of population stratification on associations of rs6232 with obesity only in the adult population [23]. Generalized linear regression was applied to test for associations of rs6232 and rs6235 with quantitative traits only in individuals without T2D. Because fasting serum insulin levels and HOMA indices were not normally distributed, they were log transformed for analysis. Interactions between the SNPs and age or gender were tested by including a two-way interaction term (SNP*age or SNP*gender) in the model. All analyses were adjusted for age and gender, and with other covariates as appropriate. The reported *P*-values are nominal and two-sided. Association analyses were performed with SPSS V15.0, statistical package; Chicago, IL.

Pairwise linkage disequilibrium (LD) between both SNPs was estimated using Haploview V3.2. (http://www.broad.mit.edu/mpg/haploview). Power calculations were performed using QUANTO software (http://hydra.usc.edu/gxe/).

## Results

The study included a total of 2382 individuals, 1176 adults and 1206 children. Minor allele frequencies (MAFs) for rs6232 and rs6235 SNPs were 0.78%, and 19.99%, respectively. Both variants are in weak linkage disequilibrium (r^2^ = 0.30). Because risk genotypes frequencies showed no significant differences in lean and overweight individuals (children or adults; *P*>0.40), both groups were considered together as non-obese subjects for the analyses. SNP rs6232 was significantly associated with obesity in children (OR = 3.78, 95%CI 1.42–9.88; *P* = 7×10^−3^) and was significantly associated with class III obesity in adults (OR = 2.61, 95%CI 1.10–6.19; *P* = 0.02,) showing a trend of association after adjusting for admixture (*P* = 0.07). The odds ratio estimated in the combined analysis was 3.01 (95%CI 1.64–5.53; *P* = 4×10^−4^, Table 2). In contrast, the rs6235 variant was not associated with obesity in children or adults (OR = 1.16, 95%CI 0.96–1.42; *P* = 0.20, in the combined analysis, Table 2). No significant genotype-age or genotype-gender interactions on the risk of obesity were observed for any SNP (P>0.05 in all tested models).

**Table 2:** Association of the rs6232 and rs6235 with obesity in children and adult populations.

rs6232 (encoding N221D)									rs6235 (encoding S690T)					
Children														
		**Genotype (%)**						**Genotype (%)**						
		**AA**	**AG**	**GG**	**G allele** **frequency**	**OR (95% CI)**	***P*** **-value**	**GG**		**GC**	**CC**	**C allele frequency**	**OR (95% CI)**	***P_add_***
**Non-obese**		795 (99.1)	7 (0.9)	0	0.44			528 (66.6)		234 (29.5)	31 (3.9)	18.66		
**Obese**		392 (97.0)	12 (3.0)	0	1.57	3.78 (1.42–9.88)	7×10^−3^	249 (62.9)		132 (33.3)	15 (3.8)	20.45	1.14 (0.92–1.43)	0.21
**Adults**														
**Non-obese**		550 (97.9)	12 (2.1)	0	1.07			347 (63.9)		172 (31.7)	24 (4.4)	20.26		
**Obese**		595 (96.9)	19 (3.1)	0	1.54	1.35 (0.63–2.91)	0.43	368 (61.5)		209 (34.9)	21 (3.5)	20.73	1.03 (0.83–1.27)	0.74
**Class I/II obese**		374 (98.4)	6 (1.6)	0	0.79	0.67 (0.24–1.85)	0.44	229 (62.2)		126 (34.2)	13 (3.5)	20.92	1.00 (0.79–1.27)	0.98
**Class III obese**		221 (94.4)	13 (5.6)	0	2.76	2.61 (1.10–6.19)	0.02	139 (60.4)		83 (36.2)	8 (3.5)	21.52	1.12 (0.84–1.49)	0.43
**Combined childhood and adult (class III) analysis**														
						3.01 (1.64–5.53)	4×10^−4^						1.16 (0.96–1.42)	0.20

Data are n (%). All odds ratios and *P*-values were calculated by logistic regression analyses using non-obese individuals as reference group, adjusting for age, sex and DT2. *P*_add_, *P*-values for the additive model.

We also explored the effect of the rs6232 and rs6235 on glucose homeostasis in non-obese and obese children and in non-diabetic adults. The rs6232 risk allele was significantly associated with lower fasting glucose levels (*P* = 0.01) and with increased insulin levels and HOMA-B (*P* = 0.05 and 0.01, respectively) only in non-obese children (Table 3). No significant associations between rs6235 and glucose homeostasis parameters were found in children or adults (Table 4).

**Table 3:** Glucose homeostasis parameters in nondiabetic children and adult populations according to *PCSK1* rs6232.

Non-obese				Obese		
Children						
	AA	AG	*P*-value	AA	AG	*P*-value
*N (1206)*	795	7		392	12	
Age (yrs)	9.42±1.92	8.33±2.25	0.70	10.26±2.29	10.23±1.90	0.97
z-score BMI	0.31±1.01	0.05±1.09	0.60	2.08±0.29	2.08±0.24	0.98
Glucose (mmol/L)	5.01±0.45	4.59±0.65	0.01	5.01±0.52	4.96±0.32	0.72
Insulin (pmol/L)	38.8 (23.6, 59.0)	52.7 (37.8, 59.0)	0.05	76.3 (48.6, 109.0)	70.1 (44.9, 117.8)	0.90
HOMA-B (%)	76.2 (57.1, 102.5)	104.2 (78.1, 122.7)	0.01	125.3 (85.3, 159.3)	118.9 (78.7, 168.2)	0.99
HOMA-S (%)	136.5 (90.5, 222.1)	101.2 (92.8, 167.8)	0.06	69.0 (49.7, 107.3)	76.9 (48.7, 120.5)	0.78
**Adults**						
*N (922)*	432	10		463	17	
Age (yrs)	45.29±14.36	41.9±22.9	0.46	40.61±12.38	36.59±15.10	0.20
BMI (kg/m^2^)	23.77±2.49	23.92±2.68	0.56	38.11±7.58	41.20±7.66	0.18
Glucose (mmol/L)	5.02±0.57	4.99±0.72	0.88	5.38±0.60	5.49±0.43	0.39
Insulin (pmol/L)	43.1 (29.9, 61.1)	42.7 (28.3, 49.3)	0.45	105.6 (69.1, 156.9)	136.1 (59.0, 164.3)	0.46
HOMA-B (%)	83.8 (63.9, 108.5)	79.8 (56.5, 98.3)	0.78	130.8 (101.6, 167.7)	132.8 (86.3, 179.0)	0.26
HOMA-S (%)	125.0 (86.2, 177.2)	125.3 (109.6, 190.8)	0.58	50.8 (34.8, 77.3)	38.5 (33.4, 94.9)	0.54

Data are means ± s.d. or medians (interquantile range). *P*-values were calculated by generalized linear regression. BMI was adjusted for age and gender. Plasma glucose/insulin levels and HOMA indices were adjusted for age, gender and BMI. HOMA-B, homeostasis model assessment of beta-cell function; HOMA-S, homeostasis model assessment of insulin sensitivity.

**Table 4:** Glucose homeostasis parameters in nondiabetic children and adult populations according to *PCSK1* rs6235.

Non-obese					Obese			
Children								
	GG	GC	CC	*P*_add_	GG	GC	CC	*P*_add_
*N (1189)*	528	234	31		249	132	15	
Age (yrs)	9.45±1.90	9.36±1.91	8.96±1.84	0.20	10.20±2.21	10.28±2.42	10.44±2.02	0.63
z-score BMI	0.32±1.01	0.28±1.00	0.35±0.89	0.88	2.08±0.27	2.09±0.31	2.07±0.22	0.72
Glucose (mmol/L)	5.04±0.47	4.95±0.40	5.05±0.53	0.16	5.03±0.51	4.98±0.55	5.04±0.28	0.71
Insulin (pmol/L)	40.2 (24.3, 59.7)	38.1 (23.6, 59.0)	45.1(21.1, 69.1)	0.77	74.3 (50.8, 106.0)	76.0 (42.5, 118.0)	85.0 (53.8,118.9)	0.17
HOMA-B (%)	76.4 (58.5, 101.0)	77.8 (56.4, 111.1)	73.3 (51.5, 97.6)	0.87	125.6 (87.5, 156.2)	112.3 (81.0, 168.1)	122.7 (101.3, 166.2)	0.16
HOMA-S (%)	135.6 (87.6, 213.2)	138.8 (90.9, 226.6)	116.0 (75.2, 245.9)	0.63	73.0 (51.9, 106.4)	69.3 (44.1, 124.4)	65.60 (45.8, 100.3)	0.34
**Adults**								
*N (893)*	267	133	22		283	169	19	
Age (yrs)	45.7±14.7	44.02±13.88	45.45±17.69	0.40	41.0±12.1	39.3±13.0	41.0±11.5	0.26
BMI (kg/m^2^)	23.7±2.4	23.92±2.53	23.83±2.55	0.31	38.5±7.8	37.8±7.6	38.6±6.56	0.40
Glucose (mmol/L)	5.7±2.3	5.58±1.91	6.15±2.38	0.86	6.0±2.2	6.1±2.1	6.3±2.7	0.59
Insulin (pmol/L)	56.2 (36.9,92.8)	62.5 (40.9, 97.9)	60.7 (42.3, 78.4)	0.23	74.6 (45.8, 132.4)	63.1 (43.05, 111.1)	89.9 (64.7, 130.5)	0.14
HOMA-B (%)	89.7 (67.6, 124.6)	95.7 (70.2, 131.6)	88.2 (58.5, 107.5)	0.79	106.7 (64.4, 141.0)	87.1 (58.0, 127.5)	111.1 (49.3, 161.5)	0.11
HOMA-S (%)	92.0 (53.9, 137.9)	83.8 (51.7, 126.5)	85.3 (66.7, 124.1)	0.24	70.9 (39.7, 116.8)	79.8 (46.6, 123.7)	61.6 (38.4, 77.8)	0.65

Data are means ± s.d. or medians (interquantile range). *P_add_*_-_values were calculated by generalized linear regression using an additive model. BMI was adjusted for age and gender. Plasma glucose/insulin levels and HOMA indices were adjusted for age, gender and BMI. HOMA-B, homeostasis model assessment of beta-cell function; HOMA-S, homeostasis model assessment of insulin sensitivity.

## Discussion

### Association with Obesity

Obesity is a complex disorder involving both genetic and environmental factors [2]. Although obesity is highly prevalent in Mexico, both in children and adults, studies on the genetic component of this disease in the Mexican population are scarce [18], [20], [24]. Recently, N221D (rs6232) and S690T (rs6235) *PCSK1* nonsynonymous polymorphisms were found to contribute to the etiology of polygenic obesity in European populations [8]. In the present case-control study, the *PCSK1* rs6235 was common but not associated with obesity in Mexican children or adults. This result is consistent with several recent studies in European and Chinese populations [9]–[14]. In contrast, rs6232 was infrequent in the Mexican population (0.78% as compared to 4–8% in Europeans), but was significantly associated with obesity in both children and adults. In fact, the association with obesity for G allele carriers was significant only in extreme phenotypes (childhood and adult class III obesity), and was higher than the risk previously reported (OR = 3.01 vs. OR = 1.34, respectively) [8]. Thus, it is likely to provide only a weak population-attributable risk for common obesity and not to be a major contributor to obesity in the general population of Mexico. However, the presence of this functional variant should be considered as a serious risk factor for extreme forms of obesity in the Mexican population, as has been recently reported for heterozygous *PCSK1* mutations in Europeans [25].

A limitation of the study was low statistical power. For the combined analysis (children and adults), the present study had only 13.6% and 78.3% statistical power to detect previously reported associations of rs6232 and rs6235 with obesity, respectively [8]. Because statistical power to detect obesity subclasses was lower, we cannot rule out the possibility of rs6235 associations with extreme forms of obesity in this population.

### Associations with Glucose Homeostasis

Both rs6235 and rs6232 have been recently associated with glucometabolic traits. Rs6235 was found to be associated with decreasing fasting glucose levels and increased HOMA-B [15], [26], while rs6232 was associated with decreased circulating post-prandial glucose and elevated glucagon levels in a Danish population-based study [27], and discordantly associated with decreased fasting insulin levels and reduced insulin sensitivity in German adults with increased risk of T2D [26]. In the present study rs6235 was not associated with glucometabolic traits in any group; however the rs6232 G-allele was significantly associated with decreased insulin sensitivity only in non-obese children. The inconsistencies of the effect of *PCSK1* variants on glucose metabolism may result from different study designs and allele frequencies, particularly in the case of rs6232 which is very infrequent in the Mexican population. Additional studies are required to confirm and further characterize the effect of these polymorphisms on glucose homeostasis.

In conclusion, although the rs6232 polymorphism is rare in the Mexican population, it is significantly associated with extreme forms of obesity. However, it is likely not to be a major contributor to obesity in the general Mexican population. Further studies with larger samples sizes are needed to confirm this association.
